# The Effect of Exercise on the Skeletal Muscle Phospholipidome of Rats Fed a High-Fat Diet

**DOI:** 10.3390/ijms11103954

**Published:** 2010-10-15

**Authors:** Todd W. Mitchell, Nigel Turner, Paul L. Else, Anthony J. Hulbert, John A. Hawley, Jong Sam Lee, Clinton R. Bruce, Stephen J. Blanksby

**Affiliations:** 1 School of Health Sciences, University of Wollongong, NSW 2522, Australia; E-Mail: pelse@uow.edu.au; 2 Garvan Institute of Medical Research, Sydney, NSW 2010, Australia; E-Mail: n.turner@garvan.org.au; 3 School of Biological Sciences, University of Wollongong, NSW 2522, Australia; E-Mail: hulbert@uow.edu.au; 4 School of Medical Sciences, RMIT University, Bundoora, Victoria 3083, Australia; E-Mail: john.hawley@rmit.edu.au; 5 Department of Physical Education, Daegu University, Daegu, South Korea; E-Mail: jslee@daegu.ac.kr; 6 Baker IDI Heart & Diabetes Institute, Melbourne, Victoria 3004 Australia; E-Mail: clinton.bruce@baker.edu.au; 7 School of Chemistry, University of Wollongong, NSW 2522, Australia; E-Mail: blanksby@uow.edu.au

**Keywords:** electrospray-ionization mass spectrometry, endurance, exercise training, fatty acids, lipidomics

## Abstract

The aim of this study was to examine the effect of endurance training on skeletal muscle phospholipid molecular species from high-fat fed rats. Twelve female Sprague-Dawley rats were fed a high-fat diet (78.1% energy). The rats were randomly divided into two groups, a sedentary control group and a trained group (125 min of treadmill running at 8 m/min, 4 days/wk for 4 weeks). Forty-eight hours after their last training bout phospholipids were extracted from the red and white vastus lateralis and analyzed by electrospray-ionization mass spectrometry. Exercise training was associated with significant alterations in the relative abundance of a number of phospholipid molecular species. These changes were more prominent in red vastus lateralis than white vastus lateralis. The largest observed change was an increase of ~30% in the abundance of 1-palmitoyl-2-linoleoyl phosphatidylcholine ions in oxidative fibers. Reductions in the relative abundance of a number of phospholipids containing long-chain n-3 polyunsaturated fatty acids were also observed. These data suggest a possible reduction in phospholipid remodeling in the trained animals. This results in a decrease in the phospholipid n-3 to n-6 ratio that may in turn influence endurance capacity.

## 1. Introduction

Biological membranes are important regulators of cellular function and increasingly it is being realized that changes in the lipid composition of membranes influence cellular function, including muscle cells [1,2]. While most studies to date have concentrated on gross changes in the total phospholipid pool that constitutes the bulk of biological membrane bilayers, electrospray-ionization mass spectrometry (ESI-MS) [3–5] has recently been used to determine the membranes constituent phospholipid molecular species (the *phospholipidome*). The data obtained by this technique can provide greater insight into the changes in membrane lipids than is possible by the analysis of the fatty acid (FA) composition of the total phospholipid pool by gas chromatography (GC). Through the use of ESI-MS, we have recently shown that exercise-induced remodeling of rodent skeletal muscle membrane at the level of phospholipid molecular species is more extensive [6] than that observed in the total phospholipid FA profile [7] in rats fed a high-carbohydrate diet. This remodeling was greater in glycolytic (white) muscle than in oxidative (red) muscle.

A number of studies have previously shown that diets high in fat increase exercise endurance of rats [8–10]. Whether a high-fat diet increases exercise endurance of humans, however, is more equivocal [11]. Here we report the results of an examination as to whether exercise modifies the phospholipidome of both red and white skeletal muscle (*vastus lateralis*) of rats fed a high-fat diet as it does in those fed a high-carbohydrate diet.

## 2. Results

### 2.1. Compliance with Training Programs and Physical Characteristics

All animals in the exercise-training groups completed each of the prescribed training sessions. There were no significant differences in the body mass of animals between the two groups at the end of the 8 wk experimental period (272 ± 10 *vs.* 271 ± 5 g). There were also no significant differences in daily energy intake between the SED and TRAINED groups, 255 ± 4 kJ *vs*. 247 ± 2 kJ respectively. The effect of exercise training intensity on endurance running capacity and enzyme activity for these rats has been reported previously [12]. In summary, the trained rats demonstrated an improved endurance capacity compared to the untrained rats (*P* < 0.05). Changes in the activities of the mitochondrial enzymes β-hydroxy-acyl-CoA dehydrogenase, citrate synthase and carnitine palmitoyl transferase were minimal [12].

### 2.2. Comparison of Oxidative and Glycolytic Fibers

**Figure 1:**
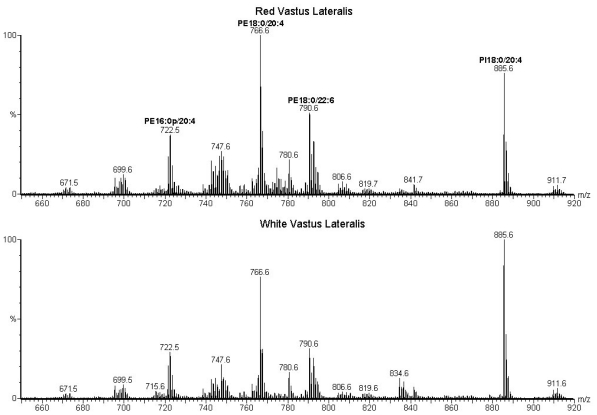
Negative ion mass spectra of phospholipid extracts from red (top) and white (bottom) vastus lateralis of a sedentary female Sprague-Dawley rat. Some of the most abundant ions have been identified. A decrease in the abundance of PI18:0/20:4 ions is clearly observed in white compared to red vastus lateralis. PE, phosphatidylethanolamine; PI, phosphatidylinositol; *m/z*, mass-to-charge ratio.

**Figure 2:**
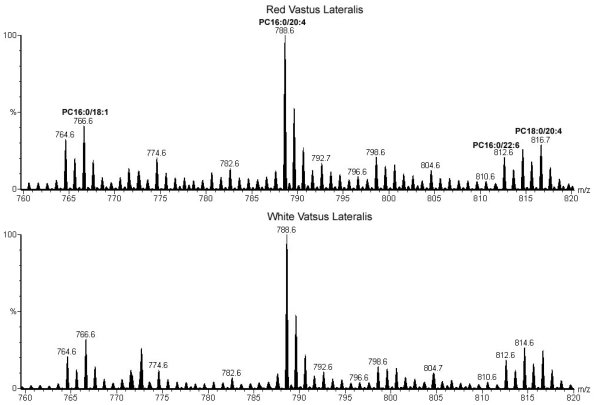
Positive ion mass spectra of phospholipid extracts from red (top) and white (bottom) vastus lateralis of a sedentary female Sprague-Dawley rat. All phospholipids are observed as [M + Li]^+^ ions. Some of the most abundant ions have been identified. PC, phosphatidylcholine; *m/z*, mass-to-charge ratio.

Representative negative ion spectra of phospholipid extracts from red and white vastus lateralis of a sedentary rat are shown in Figure 1, while Figure 2 shows positive ion spectra of the same phospholipid extracts. The major ions observed in positive and negative modes correspond to phospholipid [M + Li]^+^ and [M - H]^−^ ions, respectively. The structure of each phospholipid was determined by the characteristic fragmentations observed in the tandem mass spectrum of each ion (data not shown) [5,13]. The tandem mass spectra also revealed a number of isobaric species, for example, the negative ion observed at m/z 699.5 corresponds to a mixture of PA18:1/18:1 and PA18:0/18:2. Significantly, the same phospholipid molecular species were identified in both of the tissue types. That is, no ions were found to be unique to one tissue type. Comparison of the normalized ion abundances however, reveals significant differences in the relative concentrations of several phospholipids. The largest differences between the two negative ion mass spectra in Figure 1 are the lower relative abundance of ions at *m/z* 885.6 (PI18:0/20:4) and higher abundance of ions *m/z* 790.6 (PE18:0/22:6) in the RVL compared to the WVL.

Although the positive ion spectra in Figure 2 appear similar, some difference in normalized ion abundances are observed, e.g., a lower level of PC16:0/20:4 and higher level of PC16:0/18:1 ions in RVL compared with WVL.

A statistical analysis of muscle types from all twelve animals independent of training regime demonstrates a number of differences in the phospholipid profile between the two muscles in both the neutral (Table 1) and acidic phospholipids (Table 2). The largest differences between the two muscles were decreases in PC16:0/20:4 and PI18:0/20:4 ions and increases in PE18:0/22:6, PC16:0/18:1 and isobaric PE18:0/18:2 and PE18:1/18:1 ions in the RVL compared to the WVL. The RVL also had a higher abundance of PC16:0/18:2, PA18:1/18:2, PE16:0p/18:2, PE18:0/20:4, PC18:0/20:4, PA16:0/18:2 and the isobaric ions at *m/z* 699.5 (PA18:1/18:1, PA18:0/18:2) and *m/z* 723.5 [PA18:0/20:4 and CL(18:2)_4_]. A lower abundance of PC18:1/20:4 and PS18:0/22:6 ions was also seen in the RVL compared to the WVL.

After statistical analysis of data from all sedentary rats, four significant differences in phospholipid speciation between white and red vastus lateralis were observed. They were a lower level of PC16:0/20:4 and PI18:0/20:4 ions and a higher level of PC16:0/18:1 and PE18:0/22:6 ions in RVL. The number of differences in phospholipid profile between the two muscles increased in the training group with the additional differences being a greater abundance of PC16:0/18:2, PA18:1/18:2, PE16:0p/18:2 and the isobaric ions at *m/z* 699.5 (PA18:0/18:2, PA18:1/18:1) and *m/z* 742.6 (PE18:0/18:2, PE18:1/18:1) in RVL.

### 2.3. Comparison of Trained and Sedentary Rats

No novel molecular species were observed in glycolytic or oxidative muscles after exercise training. Nevertheless, a statistical analysis of trained verses sedentary rats, independent of muscle type, demonstrates a number of significant differences in the relative abundance of both neutral (Table 1) and acidic (Table 2) phospholipids. An increased abundance of PC16:0/18:2, PE16:0p/18:2, PA18:1/18:2 ions and the isobaric ions at *m/z* 742.6 (PE18:0/18:2, PE18:1/18:1) were all observed in the trained animals. Training was also associated with a lower abundance of PC16:0/22:6, PC18:1/20:4, PI18:0/22:6, PI18:0/22:5 and PE18:0/22:6 ions.

**Figure 3:**
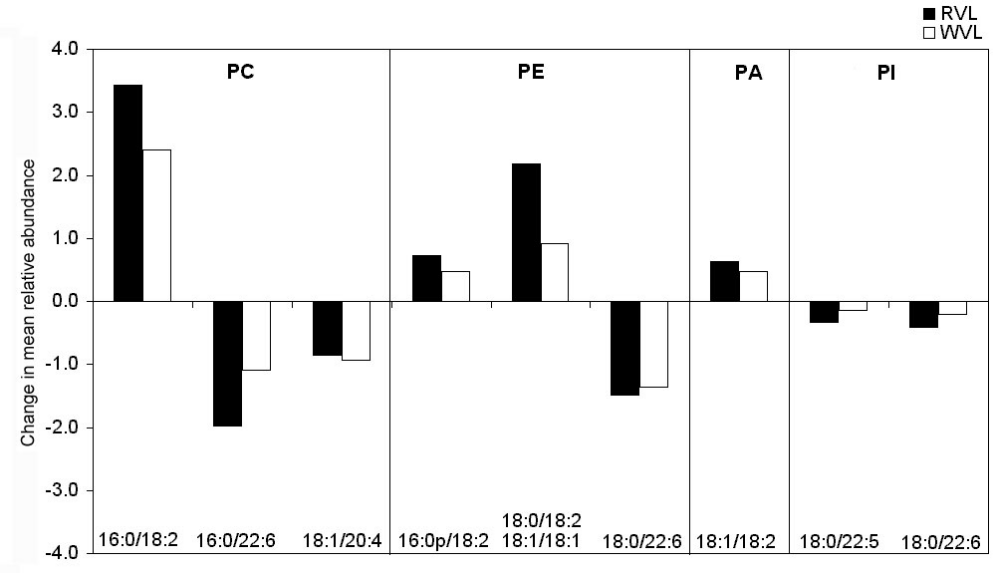
Change in mean relative abundance of phospholipid molecular species associated with exercise training in red and white vastus lateralis. Only species with an overall significant change after exercise training are displayed. PC, phosphatidylcholine; PA, phosphatidic acid; PE, phosphatidylethanolamine; PI, phosphatidylinositol. Values are expressed as the absolute change in mean normalized ion intensity from the SED group (*n* = 6 per group).

The number of significant differences within muscle type was far less. The largest effect of exercise was found to be a significantly greater abundance of PC16:0/18:2 ions in RVL. A higher abundance of the isobaric PE18:0/18:2, PE18:1/18:1 ions in the RVL training group compared to the RVL sedentary group were also apparent. Conversely, a decrease was observed in the abundance of red vastus PC16:0/22:6 ions in the trained animals. In the white vastus, however, no significant differences in the phospholipid profile were observed. The disparity in the magnitude of the observed changes between RVL and WVL can be seen in Figure 3.

## 3. Discussion

The influence of diet on skeletal muscle phospholipid FA profile is well documented [14–16]. This influence has also been reported for the rat population used in the current study [7]. It is interesting to note, however, that despite the large difference in dietary FAs, particularly the lack of essential n-3 FAs (see Table 3), the phospholipid molecular species observed are the same as those reported previously [6]. Still, a large difference in the ratio of phospholipids containing n-6 PUFAs to those containing n-3 PUFAs is apparent. Of particular interest is the higher abundance of PE18:0/22:6 in RVL when compared to WVL. Even though the abundance of this phospholipid was lower in both muscles in the current study, the ratio of PE18:0/22:6 abundance in oxidative to glycolytic muscle was almost identical to rats fed a diet with no FA deficiencies. For example, in the sedentary rats the ratio of PE18:0/22:6 ion abundance in red vastus to white vastus was 1.35 for rats fed a complete diet [6] and 1.32 for the rats fed an n-3 deficient diet in the current study. This finding reinforces previous data indicating a higher requirement of PE18:0/22:6 in RVL [6], even when there is a deficiency in dietary n-3 FA.

The relative abundance of a number of phospholipid molecular species was altered in association with exercise training. Exercise-induced changes appear to be facilitated through the variation in abundance of phospholipid species already present in the membrane and did not involve the synthesis of novel molecular species. This observation is consistent with previously reported findings [6]. Also in agreement is the finding that the largest changes occurred in PE and PC species (see Figure 2). The greatest observed effect of training on an individual phospholipid molecular species was an increase in the abundance of PC16:0/18:2 in oxidative muscle. This phospholipid is one of the major *de novo* synthesized PCs and is the precursor of other PC species that are created through its deacylation/reacylation [17]. The concomitant reduction in PC16:0/22:6 may be the result of a reduced conversion of PC16:0/18:2 to the former species during exercise. This would likely be exacerbated by the lack of available n-3 PUFAs from the diet and may explain why this effect was found to be larger in the current animal population than reported previously [6].

The overall trend in phospholipid molecular species profile appeared to be (i) decreases in PC, PE and PI species containing long-chain PUFAs, particularly n-3 PUFAs and (ii) increases in their shorter-chain 18:2 n-6 counterparts with exercise (Figure 3). It appears, therefore, that low intensity exercise training results in an increased ratio of n-6 to n-3 PUFAs in oxidative skeletal muscle of high-fat fed rats. There is a common link here in that both exercise training and diet increased endurance capacity and reduced the n-3 to n-6 ratio in the skeletal muscle of the rats used in this study [7,12]. Other work also suggests that decreasing the ratio of n-3 to n-6 PUFAs in rat skeletal muscle can increase their endurance [18] and that n-6 content of mammalian skeletal muscle is associated with increased running speed [19]. The current finding that RVL is affected to a greater extent than WVL in the high-fat fed animals is in contrast to previous data from high-carbohydrate fed animals [6]. This suggests that under the influence of the current diet there is a greater involvement of the oxidative muscle’s membrane during exercise, which would be consistent with a role in endurance capacity. Interestingly, short-term high fat feeding has previously been shown to increase gene expression of enzymes involved in the uptake and oxidation of fatty acids (fatty acid translocase and β-hydroxylacyl-CoA dehydrogenase) in the skeletal muscle of male endurance athletes [20]. It is unknown if this effect is more pronounced in oxidative than glycolytic muscle.

## 4. Experimental Section

### 4.1. Animal Care, Dietary Treatments and Exercise-Training Program

Twelve female Sprague-Dawley rats with an initial body mass of 90–100 g were obtained from the Animal Resource Centre, Monash University, Melbourne, Victoria, Australia and housed two per cage. Rats were kept at a constant 22 ± 1 °C and 50 ± 2% relative humidity, with a 12 hour light-dark cycle (light 0700–1900 h). The rats were fed a diet similar in macronutrient content to previous studies examining the effect of a high-fat diet on endurance capacity [8,9,21] (78.1 E% fat, 21.9 E% protein and 0 E% carbohydrate). The FA composition of the diet was determined using GC as previously described [16] and is presented in Table 3. All experimental procedures were approved by the Animal Experimentation Ethics Committee of RMIT University.

In the first week of the training protocol, all animals were familiarized with exercise by running on a motorized treadmill at 16 m min^−1^ for 10 min day^−1^ on a custom-built eight lane motorized treadmill in the hours before dark. The treadmill was not equipped with any form of electric shock device. After one week, the rats were randomly divided into two groups, a control sedentary group (SED, n = 6) that performed no specific training and an exercise trained group (TRAINED, n = 6). The trained group had exercise intensity gradually increased over 4 weeks until they could complete 1000 m of treadmill running 4 days wk^−1^. They then performed 125 min of running at 8 m min^−1^, 4 days wk^−1^. This program was chosen because previous investigations have shown this speed to elicit ~45% of maximal O_2_ uptake in rats, an intensity at which lipid oxidation predominates over carbohydrate oxidation [22]. This was an important factor in our original study examining the effect of high-fat and high-carbohydrate diets on endurance [12].

### 4.2. Animal Sacrifice and Tissue Preparation

After the 8 wk training program, animals were euthanized by heart removal under anesthesia [intraperitoneal injection of sodium pentobarbital at 60 mg (kg body weight) ^−1^] 48 h after their last training bout. The muscles from the right hind limb were exposed and the red *vastus lateralis* (RVL, 16% type I, 33% type IIa, 50% type IIb fibers) and white *vastus lateralis* (WVL, 100% type IIb fibers) [23] were dissected out, frozen in liquid nitrogen and then stored at −80 °C until analyzed.

### 4.3. Phospholipid Extraction

All solvents used in the lipid analysis were of ultra-pure grade, purchased from Merck Pty Ltd (Kilsyth, Vic, Australia) and Crown Scientific (Moorebank, NSW Australia). Analytical grade butylated hydroxytoluene was purchased from Sigma Aldrich (Castle Hill, NSW, Australia). Skeletal muscle lipids were extracted by standard methods [24] using ultra-pure grade chloroform:methanol (2:1 v/v) containing 0.01% butylated hydroxytoluene as an antioxidant. Phospholipids were separated by solid phase extraction on Strata SI-2 silica cartridges (Phenomenex, Pennant Hills, NSW, Australia).

### 4.4. Mass Spectrometry

ESI-MS analyses were performed on a Micromass Q-ToF2 (Micromass, Manchester, UK) equipped with an electrospray ion source. All ESI-MS and ESI-MS/MS analyses were performed as previously described [6]. In brief, cone voltage was set to 70 V for negative and 30 V for positive ions. The capillary charge was set to 2800 V and MCP at 2300 V. The source was heated to 80 °C and desolvation temperature set to 120 °C. Phospholipids were diluted to 25 μM in methanol:chloroform (2:1, v/v). For negative ion analysis, the pH of the sample was increased to 10 by the addition of ammonia. To facilitate the formation of lithium adducts for positive ion characterization, lithium iodide was added (~20 nmol μL^−1^). Positive ion analysis was used to monitor neutral phospholipids while negative ion analysis was used for acidic phospholipids. Samples were infused (20 μL min^−1^) using a Harvard syringe pump and phospholipids were detected in the *m/z* range of 650 to 920. Typically, 100–120 spectra were averaged for each phospholipid extract. ESI-MS/MS spectra were obtained using argon as the collision gas at energies ranging from 32 to 45 eV.

Each mass spectrum was normalized as a percentage of the total phospholipid ions observed within the mass range after correction for isotope contributions. Although absolute phospholipid concentrations cannot be determined by this method, owing to differences in the ionization efficiency of the different head groups, relative changes in individual phospholipid molecular species between exercise groups and fiber types were assessed. This approach is analogous to the shotgun approach of Han and Gross [4,13,25].

Phospholipid nomenclature is presented as the two-letter acronym of the head group, *i.e.*, phosphatidylcholine (PC), phosphatidic acid (PA), phosphatidylglycerol (PG), cardiolipin (CL), phosphatidylserine (PS), phosphatidylinositol (PI) and phosphatidylethanolamine (PE) followed by the two FA chains (four in the case of CL). The letter p after the first FA represents a plasmenyl (vinyl ether linked) FA.

### 4.5. Statistical Analysis

Data analysis was performed using a two-way analysis of variance (ANOVA) with training or muscle type as fixed factors. Where ANOVA revealed a significant effect, Tukey’s post hoc test was administered to identify differences between training groups. Significance was accepted at the level of *P* < 0.05 and results are presented as means ± S.E.M. All statistical analyses were performed using JMP version 4.0 statistical software (SAS Institute Inc., Cary, USA).

## 5. Conclusions

In summary, under the influence of the high-fat diet used in the current study, exercise had a greater effect on the phospholipidome of oxidative than that of glycolytic muscle. This is in contrast to the effect of exercise in high-carbohydrate fed rats. The largest difference in phospholipid profile was an increase in the level of *de novo* created phospholipid species and a decrease in species created through their remodeling in trained compared to sedentary rats. This may be a result of exercise training’s influence on associated enzyme activity, *i.e.*, phospholipases A, transacylases and acyltransferases, possibly through a change in substrate availability as β-oxidation increases.

## Figures and Tables

**Table 1:** Neutral phospholipid molecular species.

Molecular species	*m/z* [M + Li]^+^	SED		TRAINED		*P* Value	

		WVL	RVL	WVL	RVL	T	M
PC16:0/18:1	766.6	12.5 ± 0.3	15.4 ± 0.5†	12.3 ± 0.6	14.4 ± 0.3†	NS	<0.0001
PC16:0/18:2	764.6	10.4 ± 0.4	12.6 ± 0.3	12.8 ± 0.9	16.1 ± 1.0*†	0.0005	0.0007
PC16:0/20:4	788.6	43.4 ± 0.7	36.0 ± 1.0†	42.6 ± 0.9	36.0 ± 0.4†	NS	<0.0001
PC16:0/22:6	812.6	8.3 ± 0.4	8.8 ± 0.5	7.2 ± 0.3	6.8 ± 0.3*	0.0006	NS
PC18:0/18:2, PC18:1/18:1	792.6	6.0 ± 0.5	8.0 ± 0.3	7.6 ± 1.8	8.5 ± 0.3	NS	NS
PC18:0/20:4	816.6	9.4 ± 0.4	10.0 ± 0.3	8.4 ± 0.8	10.1 ± 0.2	NS	0.02
PC18:1/20:4	814.6	10.1 ± 0.3	9.1 ± 0.1	9.2 ± 0.7	8.2 ± 0.3	0.04	0.02

*m/z*, mass-to-charge ratio; M, molecular mass; Li, lithium; T, training; SED, sedentary; TRAINED, low intensity training (8 m min^−1^); M, muscle; WVL, white vastus lateralis; RVL, red vastus lateralis; NS, not significant.

* Significantly different from SED (P < 0.05).

† Significantly different from WVL (P < 0.05). Values are expressed as mean normalized ion intensities ± S.E.M. after correction for isotope contributions (n = 6 per group).

**Table 2:** Acidic phospholipid molecular species.

Molecular species	*m/z* [M − H] ^−^	SED		TRAINED		*P* Value	

		WVL	RVL	WVL	RVL	T	M
PA16:0/18:2	671.5	1.1 ± 0.0	1.2 ± 0.0	1.2 ± 0.0	1.5 ± 0.1	NS	0.05
PA16:0/20:4, PA18:2/18:2	695.5	2.6 ± 0.1	2.8±0.1	2.5 ± 0.1	2.6 ± 0.1	NS	NS
PA18:0/20:4, CL(18:2)_4_	723.5	0.5 ± 0.0	0.9 ± 0.1	0.3 ± 0.0	0.9 ± 0.0	NS	0.005
PA18:0,18:2, PA18:1/18:1	699.5	2.0 ± 0.0	2.5 ± 0.1	1.9 ± 0.1	2.5 ± 0.1†	NS	0.0007
PA18:0/22:6, PA18:1/22:5, PG16:0/18:1	747.6	6.5 ± 0.1	6.2 ± 0.1	6.9 ± 0.1	6.2 ± 0.1	NS	NS
PA18:1/18:2	697.5	1.4 ± 0.1	2.1 ± 0.1	1.9 ± 0.1	2.8 ± 0.1†	0.02	0.002
PE16:0p/18:2	698.5	1.3 ± 0.0	1.9 ± 0.1	1.8 ± 0.1	2.6 ± 0.1†	0.006	0.003
PE16:0p/20:4	722.5	9.2 ± 0.2	9.1 ± 0.2	9.9 ± 0.4	9.6 ± 0.4	NS	NS
PE16:0/22:6, PS16:0/18:0	762.6	2.6 ± 0.1	2.4 ± 0.1	2.4 ± 0.0	2.3 ± 0.1	NS	NS
PE18:0/18:2, PE18:1/18:1	742.6	3.9 ± 0.1	5.4 ± 0.1	4.9 ± 0.1	7.6 ± 0.3*†	0.0005	<0.0001
PE18:0/20:4	766.6	23.1 ± 0.4	24.8 ± 0.5	24.2 ± 0.2	27.0 ± 0.2	NS	0.02
PE18:0/22:6	790.6	10.1 ± 0.2	13.4 ± 0.4†	8.9 ± 0.2	12.0 ± 0.2†	0.02	<0.0001
PS18:0/22:6	834.6	3.6 ± 0.2	2.3 ± 0.2	2.8 ± 0.2	1.9 ± 0.2	NS	0.05
PI18:0/20:4	885.6	29.2 ± 0.4	21.7 ± 0.5†	27.9 ± 0.6	18.3 ± 0.9†	NS	<0.0001
PI18:0/22:5	911.6	1.4 ± 0.0	1.5 ± 0.1	1.3 ± 0.0	1.1 ± 0.0	0.04	NS
PI18:0/22:6	909.6	1.5 ± 0.0	1.7 ± 0.1	1.3 ± 0.0	1.3 ± 0.0	0.02	NS

*m/z*, mass-to-charge ratio; M, molecular mass; H, hydrogen; T, training; SED, sedentary; TRAINED, low intensity training (8 m min^−1^); M, muscle; WVL, white vastus lateralis; RVL, red vastus lateralis; NS, not significant.

* Significantly different from SED (P < 0.05).

† Significantly different from WVL (P < 0.05). Values are expressed as mean normalized ion intensities ± S.E.M. after correction for isotope contributions (n = 6 per group).

**Table 3:** Fatty acid composition of diet.

Fatty acid	%Total
16:0	17.9
16:1 (n−7)	20.9
18:0	19.7
18:1(n−9)	35.1
18:2 (n−6)	6.4
